# Maternal and neonatal outcomes for women giving birth after previous cesarean

**DOI:** 10.18332/ejm/108297

**Published:** 2019-04-17

**Authors:** Anastasia Charitou, Dimitrios Charos, Iliana Vamenou, Victoria G. Vivilaki

**Affiliations:** 1 Department of Midwifery, University of West Attica, Athens, Greece

**Keywords:** outcomes, Vaginal Birth after Cesarean, VBAC, trial of labour, TOL, repeat cesarean section

## Abstract

**INTRODUCTION:**

Rising rates of caesarean section (CS) is an issue of particular concern. Recently, there has been research supporting Vaginal Births After Caesarean (VBAC), which is controversial. In Greece, over half of births in the country are by CS, placing Greece among countries with the highest CS rates. The aim of this study was to investigate the prevalence and the factors associated with VBACs and to compare the maternal/neonatal outcomes with a ‘non-caesarean’ control group.

**METHODS:**

The data were evaluated and retrospectively gathered on archived singleton births, from medical records of a midwifery-led team, between May 2006 and May 2013. The target group of the study included mothers with a previous CS, who had a second birth. The sample consisted of 71 VBAC women and 583 who had normal spontaneous vaginal delivery (NSVD) as the ‘non-caesarean’ control group.

**RESULTS:**

The duration of labour was longer for the VBACs compared with first-time mothers who gave birth naturally (for duration 481–720 min, 27% vs 10.3%, respectively), episiotomy was more common for VBAC (20.7% vs 7.9%), and epidural analgesia was more often for VBAC (68.4% vs 10%). The percentage of 1-min Apgar score in the range 0–7 in the VBAC group was 5%, and there was no significant difference in women who had NSVD (3.6%). The Apgar score in the 5th minute was always above 8 for both groups.

**CONCLUSIONS:**

Severe maternal and neonatal complications are infrequent, and therefore the necessity arises for further continuous studies to ascertain the safety of VBAC.

## INTRODUCTION

Rising rates of caesarean section (CS) is an issue of particular concern in the global maternity care field^1^ due to the increased adverse maternal and neonatal outcomes associated with CS^2-5^. In 1985, the World Health Organization (WHO) suggested that there are no additional advantages of CS above a rate of 10–15%^6-10^. CS rates are increasing in both resource-intense and resource-poor countries; however, of concern is the variation in CS rates internationally. For example, Europe, Netherlands, Slovenia, Finland, Sweden, Iceland and Norway have rates below 20%, whereas Italy and Cyprus have national CS rates of 38% and 52%, respectively^11^. In Greece, the CS rate has been constantly rising over the years. Over half of the births in the country are by CS, placing Greece among the countries with the highest CS rates in the world^12^. Repeated CSs constitute a significant number.

The question whether a birth after a previous CS should also be delivered by CS has been debated by experts for decades. The phrase ‘once a caesarean, always a caesarean’ has been repeated and supported frequently. However, recently, there has been a motion towards encouraging and supporting VBAC^13^. Concerns about the increasing CS rate have resulted in a consensus statement by the American College of Obstetricians and Gynecologists (ACOG) that ‘most women with one previous caesarean delivery with a low-transverse incision are candidates for and should be counseled about VBAC’^14,15^. Repeated elective caesarean section (ERCS) is associated with an increased risk of complications such as: increased blood loss, blood clots, abdominal organ injury, infection, placenta praevia, placenta accrete, and hysterectomy^4^. Babies born by CS are at risk of respiratory distress syndrome^16^, persistent pulmonary hypertension and admission to a neonatal intensive care unit^17,18^. Previous CS has also been associated with an increased risk of stillbirth^19^.

One uncommon, but potentially serious complication associated with prior uterine surgery, including previous CS, is that of uterine rupture. This may occur prior to the onset of labour or during labour while a woman is undergoing a planned vaginal birth after caesarean (VBAC)^20^. This complication can be life-threatening for both the woman and her baby. The risk of scar rupture following VBAC has been estimated from large prospective studies to vary from 0.2%^21^ to 0.7%^22^. Maternal mortality has been shown, through a systematic review and meta-analysis of 203 research reports to be significantly increased with ERCS compared with elective VBAC (1.34 vs 0.38 per 10000)^3^. A previous successful vaginal birth is considered a protective factor, decreasing the probability of uterine rupture regardless of whether it happened before or after the previous caesarean^23^. The success rate reported varies from 49% to 87%^3^. The strongest factor for a successful VBAC is having a successful vaginal birth before or after a CS^24^. Two or more previous caesarean births have traditionally justified ERCS, however, research has indicated no significant differences among VBAC success rates (75% vs 70%), uterine rupture (0.7% vs 1.6%) and hysterectomy (0.2% vs 0.5%) between women who had a single prior caesarean compared with those who had two previous caesarean deliveries^25^.

Therefore, planned VBAC is supposed to be safe and suitable for most women who experienced one previous caesarean delivery and for some women who have had two prior caesarean deliveries, giving them the opportunity of trial of labour, depending on whether they have prognostic factors for successful VBAC (e.g. a previous vaginal birth). This point of view is supported by ACOG (2010) and National Institute for Health and Care Excellence (NICE) (2011)^15,25,26^.

Health care professionals should inform women that they should have no restrictions concerning analgesia options. Available data indicate that epidural analgesia does not have a negative impact on the success rate of VBAC^23,25^.

Oxytocin can be used for induction of labour in hospitalized patients when the cervix is ripe. In a study of 142075 attempted VBACs where oxytocin was used in 43% of cases the uterine rupture rate was 0.62%. A slightly increased rupture risk was reported for the use of oxytocin for induction compared to augmentation of labour (1.1% vs 0.8%). An unripe cervix (Bishop score <6) significantly increases the rupture risk. The use of prostaglandins before oxytocin administration is associated with a higher rupture risk (1.40–2.24%) than that of oxytocin alone^27^.

Induction of labour (especially in women with immature cervix or by prostaglandin methods) or augmentation of VBAC are related with a 2-3-fold increased risk of uterine rupture and about 1.5-fold increased risk of caesarean delivery compared to spontaneous VBAC birth^25^. The use of Foley catheters to induce labour does not appear to be associated with an increased rate of uterine rupture^25,28^.

A systematic review focusing on ways of increasing VBAC found that the use of decision aids and information programs do not have a significant effect on VBAC rates. Nevertheless, decision-aids and information programs significantly decrease women’s indecision about the mode of birth, significantly increase their knowledge on the risks and benefits of the possible modes of birth and consequently they are of value5 for hospitals^29^.

This study aims to compare perinatal indicators among women who have attempted VBAC and those of women who give birth by NSVD, to demonstrate that VBAC is a safe delivery method, and thus should be proposed as a first choice to women with previous CS, if they fulfill all requirements.

## METHODS

The data of the survey were evaluated and retrospectively gathered on archived singleton births from medical records of a midwifery-led team (cephalic presentation, >36/0 weeks of pregnancy). Data collection was achieved by a retrospective cohort study. The study took place in 2013 and the medical records of women were from May 2006 to May 2013. The target group of the study included mothers with a previous CS who had a second birth. The evaluation was conducted as a two-group comparison. The sample consisted of 71 women who had VBACs and 583 women who had NSVD as the ‘non-cesarean’ control group. Ethics approval was obtained from the midwifery center in which the midwifery-led team works. All the information and data resulting from births were used only for research purposes and absolute anonymity was maintained.

A questionnaire was developed according to the objectives and research questions of the study in order to maintain the sequence and uniformity of data. The questionnaire included questions concerning sociodemographics, obstetric history status, delivery of the placenta, newborn characteristics, breastfeeding, and if they had attended prenatal classes and for how long. The questionnaire was completed by the research team using the data from women’s medical records. The questionnaire was built only to help organize data from the medical records and so was not validated. Most medical records were complete, but some data were missing from a very small number of medical records.

The SPSS 12 software package was used for evaluating the data based on simple statistical measures of descriptive statistics and for the better analysis of the data. Inductive methods, such as the χ^2^ test were also used. The level of statistical significance was set at p<0.05.

## RESULTS

The majority of women were married (97.5%), while unmarried were 1.8% and women unmarried but with a partner were 0.3%. The majority were 31–40 years old (60.6%). Most women gave birth at 39–40 (31.6%) weeks of pregnancy, followed by those who gave birth at 40–41weeks (25%) and those at 38–39 weeks (24%). Α small percentage had between 37–38 weeks of gestation (Table 1).

**Table 1:** The configuration of the main variables of the study

*Variables*	*Results (%)*
**Marital status**	
Married	97.5
Unmarried	1.8
Unmarried with a partner	0.3
Missing data	0.4
**Age** (years)	
31–40	60.6
**Week of labor**	
39–40	31.6
38–39	24
40–41	25
**Method of delivery**	
NSVD	80.6
VBAC	9.8
CS	4.4
Vaginal birth with ventouse	4.7
Missing data	0.5
**Type of childbirth**	
Birth center	32
Hospital	17.4
Hospital like home	9.5
Birth in water	7.1
Forceps	4.8
Home birth	25.9
Missing data	3.3
**Birth duration** (min)	
121–480	66.6
0–120	16.2
481–720	10.7
>721	6.5
**Onset of labour**	
Spontaneous onset of labour	95.6
Induced labour	1.8
Missing data	2.6
**Placental expulsion**	
Automatic	90.5
**Placenta**	
Intact	98.8
Fragmented	1.2
**Perineum**	
Intact perineum	39.4
Needed episiotomy	12.3
1st degree tear	23
2nd degree tear	9.4
Frenulum rupture	10.7
**Analgesia**	
Epidural analgesia	29
Water and movement	18.1
Water	15.5
Homeopathy	14.8
Combination of water /massage /breathing etc.	10.3
**Sex of babies**	
Boys	50.4
Girls	40.1
Twins	9.5
**Weight of babies (g)**	
3000–3500	47.1
3500–4000	23.3
2500–3500	20.7
>4000	6.1
1500–2500	2.7
**Apgar score** (at 1min)	
07–Oct	98.9

**Figure 1:**
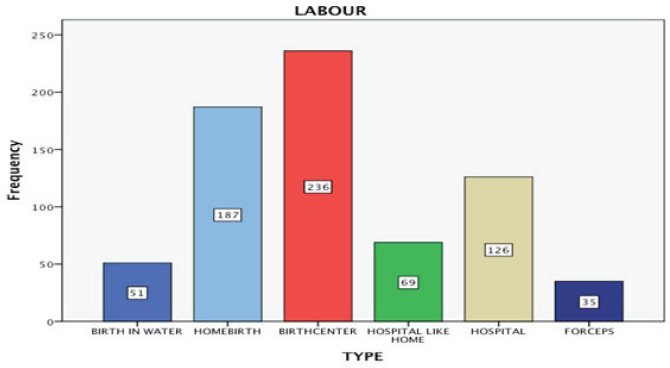
Type of childbirth

The method of delivery in the vast majority was NSVD (80.6%), followed by VBAC (9.8%), vaginal birth with ventouse (4.7%), and CS (4.4%). The place of childbirth varied among the population studied. Most women gave birth in a birth center (32.6%), whereas a significant percentage gave birth at home (25.9%) (Figure 1).

A smaller percentage gave birth at hospital (17.4%), while some gave birth in a ‘home-from-home’ delivery room and some had a waterbirth (7.1%). The labour was spontaneous for 95.6% of the women (automatic onset of labour), while 1.8% had a scheduled induced labour.

The duration of labour lasted 121–480 min for most women (66.6%), while for 16.2% it lasted 0–120 min, for 10.7% it was 480–720 min and for 6.5% the duration was >720 min.

The placental expulsion was normal for 90.5%, while manual extraction or endometrial ablation was needed for 0.3% of women. The placenta was intact for 98.8%, whereas 1.2% had fragmented placenta. Most of women (39.4%) had an intact perineum, 12.3% needed episiotomy, 23% had 1st degree tear and 9.4% had 2nd degree tear of the perineum, and 10.7% had frenulum rupture. Epidural analgesia was used by 29% of the women, whereas smaller percentages used water and movement, used only water, used homeopathy, used combination of movement/massage/breathing, used analgesics (pethidine, fentanyl) or a combination of water/movement/homeopathy (18.1%, 15.5%, 14.8%, 10.3%, 3.9% and 1.3%, respectively). It is also very interesting that prenatal classes were attended mostly by women that gave birth naturally.

Baby boys were slightly more than girls with a small difference (50.4% boys, 40.1% girls). Most babies weighed 3000–3500 g (47.1%). The next category was 3500–4000 g (23.3%), while there was a large percentage of newborns that weighed 2500–3500 g (20.7%). Some of the newborns weighed >4000 g while others were 1500–2500 g (6.1% and 2.7%, respectively). Newborns’ length was mainly 50–55 cm (64.5%). A significant percentage had length between 45–50 cm (31.5%) and a very small percentage was >55 cm (4.3%). Apgar score in the 1st minute was 4–6 in only 4 newborns (1.1%), while most babies had 7–10 (98.9%). There were no differences in the Apgar score in 5 minutes as most babies had 7–10 from the first.

Table 2 compares the results of the VBAC group with those of NSVD group. There was a statistically significant difference in the duration and the way of birth in the group of women who gave birth in 481–720 min between those who had a VBAC (27%) and the first-time mothers who gave birth naturally (10.3%) (χ^2^=1130.789, df=618, Exact. Sig. (2-sided) = 0.2). Furthermore, episiotomy rates were significantly higher in women who had VBAC (20.7%) than for the first-time mothers who gave birth naturally (7.9%) (χ^2^=645.273, df=15, Exact. Sig. (2-sided) = 0.1). The study showed that epidural analgesia was more common in VBAC (68.4%), while 10% of the first-time mothers used epidural (χ^2^=80.657, df=21, Exact. Sig. (2-sided) = 0.1).

**Table 2:** Comparing results of VBAC group with NSVD group

*Variables (N)*	*NSVD (N)*	*VBAC (N)*	*p*
**Family status**			
Married	567	70	0.966
Partners	2	0	
Single mother	11	1	
**Method of delivery**	583	71	0.000
**Type of childbirth**			
Birth center	236	0	0.000
Home birth	187	0	
Hospital	126	64	
**Birth**			
Induction of labour	1	1	0.000
Spontaneous onset of labour	576	67	
**Placental expulsion, non-automatic**	6	0	0.901
**Fragmented placenta**	6	1	0.569
**Perineum**			
Episiotomy	40	12	0.000
1st grade rupture	129	11	
2nd grade rupture	53	3	
Frenulum rupture	56	10	
Caesarean section	0	7	

There was a borderline non-statistical difference between Apgar score in 1st minute and way of birth. The observed level of statistical significance was 5=5%, which means that in the VBAC group the percentage of newborns who had Apgar score between 0–7 was 5% and consequently non-significant different from the percentage of the primiparous who gave birth naturally (3.6%) (χ^2^=12.999, df=3, Exact. Sig. (2-sided) = 5).

There were no statistical differences between the way of birth and each one of: age of the women, family status, placental expulsion, placenta, or birth weight.

Significant differences were evaluated (p<0.05) between VBACs and primiparous who had a vaginal delivery in the variables: maternal age (χ^2^=70.173, df=78, Asymp. Sig.= 0.632), duration of labour (χ^2^=41.739, df=9, Asymp. Sig.= 0.000 <0.05), family status (χ^2^=1.399, df=6, Asymp. Sig. = 0.966), perineum (χ^2^=645.273, df=15, Asymp. Sig. = 0.000 <0.05), birth weight (χ^2^=8.207, df=15, Asymp. Sig. = 0.826), analgesia (χ^2^=80.657, df=21, Asymp. Sig. = 0.000 <0.05), and Apgar Score in 5 min ≤7 (χ^2^=12.999, df=3, Asymp. Sig. = 0.095).

Finally, in the study there was no perinatal death incident and the risk of uterine rupture was zero. Perineum surgery was not required for 12 of the 58 cases. Although, there were several cases of frenulum rupture (10/58), episiotomy (12/58), and first grade rupture (11/58).

## DISCUSSION

This study was conducted in order to highlight VBAC as a safe delivery method. In addition, in recent years, an increasing number of studies confirm that VBAC is a safe way for women to give birth and claim that healthcare professionals should suggest it more often.

The aim of our study was to collect and analyze the data on the birth outcomes for women with a previous caesarean. The study was conducted on a population of 720 women. The results of the study were compared with the results of other research in order to ascertain similarities and differences. Concerning primiparous women and VBAC, the duration of labour appears longer for the VBACs (27% of VBACs were in the group of 481–720 min compared with 10.3% primiparous women). Apgar Score in 1st minute has an important role in the postnatal condition of the newborn. In the VBAC group, 5% newborns had an Apgar score between 0–7. Consequently, a non-significant percentage of the primiparous women gave birth naturally. Apgar Score in the 5th minute for both groups was always above 8.

There was no significant difference between the ages of primiparous women who gave birth vaginally and the women who had VBAC. These findings are in agreement with the findings of a German study^13^. Furthermore, there was no significant difference between the week of pregnancy and the VBAC, as for both groups (VBAC and primiparous) birth took place mostly in the 39–40 week. These findings are in agreement with the findings of the German study, in which there was also no statistical difference^13^. Epidural analgesia is more often performed in VBACs (68.4% vs 10%) according to our study, which is in agreement with that of a recent study^30^. Epidural analgesia does not conceal the signs and symptoms related to rupture of the uterus and are not contraindicated in VBAC labour. In fact, epidural analgesia has been linked with improved rates of VBAC success, compared to non-receiving women. However, epidural analgesia may raise the risk of delayed second stage and operative vaginal birth^25^.

We found that the percentage of spontaneous labour was 95.6%. These findings are opposed to the data of the German study^13^, which showed that automatic onset of labour occurred in 73.5%. In addition, the success rate of other studies was 72%^24^, 73%^33^ and 73.3%, i.e. there were 11266 successful VBACs in 15323 attempts^34^. A lower percentage (60–80%) for VBAC is mentioned by another study^31^.

Episiotomy rates were 20.7% in the VBAC group compared with 7.9% in first-time mothers. However, a study which took place in Jordan found that primiparous women had higher rates of episiotomy (reaching nearly 100%), which is opposed to our findings^32^. It is a very important fact that in our study, in all cases of VBAC the placental expulsion was normal and manual extraction or endometrial ablation was not needed. The placenta also was intact almost in 100% of cases, and only in one case it was fragmented, while in 6.8% primiparous women the placenta was fragmented. These findings are in agreement with the Jordan study^32^, which found that primiparous women exhibited higher levels of manual extraction of the placenta. According to another study, the previous VBAC does not compound the risk of uterine rupture during the next attempt. There are some studies, however, which claim that the previous VBAC relates to reduced risk of uterine rupture^24^.

There was no difference between maternal and neonatal mortality between the two groups, as the percentage was zero for both. These findings are in agreement with the German study^13^, in which there was no significant statistical difference between maternal and neonatal mortality rates. Another research showed similar results, since it did not find an increase in perinatal death in women who had a trial of labour after CS^22,33,35^. Furthermore, a different study compared the results between women who chose VBAC method and those who selected repeated CS (ERCD) and found that maternal mortality remains a very rare event regardless of the delivery method. Also, maternal morbidity is mainly due to the failure of a ‘normal’ birth effort (Trial of Labor – TOL) and the risks of unplanned caesarean section during childbirth. As for the total rupture of the matrix, its percentages increase remarkably with TOL in comparison with ERCD but still remain at very low levels (about 0.2–0.8%) for women who have only one incision. The incidents of complications and injuries, such as bladder problems, bleeding, venous thromboembolic complications, infection, etc. are independent of the type of delivery^27^.

Moreover, the birth weight did not show a difference between the two groups, which is again in agreement with the results of the German study^13^.

Additionally, rates of VBAC were relatively low (9.8%). This proportion is lower than the target the Health Services have set. It has to be noted however, that this target was 35% until 2000, which has not been achieved, while at the same time repeated caesarean sections increased in the USA^22^. At least, half of the women who delivered with VBAC gave birth in a hospital. Regarding the type of anesthesia, few (13) were administered an epidural sedation.

### Limitations

Due to the small number of participants in our study, it is difficult to make generalizations to other populations. Naturally, the survey is slightly incomplete as it was not possible for all the necessary information to be gathered. Future studies may include other variables such as infant respiratory problems, potential injuries of the newborn, prematurity, fetal and maternal mortality, bleeding after the birth, placenta previa or placenta retention. Another limitation is that the questionnaire, used for the organization of the data, was not validated. Also, there were some missing data from the medical records.

## CONCLUSIONS

Generally, it is indicated that VBAC constitutes a safe method of delivery and is considered necessary for pregnant women who have at least one CS to be informed, advised and motivated to have a vaginal delivery. A high percentage of previous VBACs are linked to a greater likelihood of VBAC success, as well as a lower risk of rupture of the uterus and perinatal complications in the present gestation. Severe maternal and neonatal complications exist but are infrequent, nevertheless, further studies to ascertain the safety of VBAC are warranted.
